# Abrupt elastic-to-plastic transition in pentagonal nanowires under bending

**DOI:** 10.3762/bjnano.10.237

**Published:** 2019-12-12

**Authors:** Sergei Vlassov, Magnus Mets, Boris Polyakov, Jianjun Bian, Leonid Dorogin, Vahur Zadin

**Affiliations:** 1 Institute of Physics, University of Tartu, W. Ostwaldi Str. 1, 50412, Tartu, Estonia; 2 ITMO University, Kronverskiy pr., 49, 197101 Saint-Petersburg, Russia; 3 Institute of Solid State Physics, University of Latvia, Kengaraga 8, LV-1063, Riga, Latvia; 4 Department of Industrial Engineering, University of Padova, Via Gradenigo 6/a, 35131 Padova, Italy,; 5 Institute of Technology, University of Tartu, Nooruse 1, 50411 Tartu, Estonia

**Keywords:** finite element method, mechanical properties, molecular dynamics, nanowires

## Abstract

In this study, pentagonal Ag and Au nanowires (NWs) were bent in cantilever beam configuration inside a scanning electron microscope. We demonstrated an unusual, abrupt elastic-to-plastic transition, observed as a sudden change of the NW profile from smooth arc-shaped to angled knee-like during the bending in the narrow range of bending angles. In contrast to the behavior of NWs in the tensile and three-point bending tests, where extensive elastic deformation was followed by brittle fracture, in our case, after the abrupt plastic event, the NW was still far from fracture and enabled further bending without breaking. A possible explanation is that the five-fold twinned structure prevents propagation of critical defects, leading to dislocation pile up that may lead to sudden stress release, which is observed as an abrupt plastic event. Moreover, we found that if the NWs are coated with alumina, the abrupt plastic event is not observed and the NWs can withstand severe deformation in the elastic regime without fracture. The coating may possibly prevent formation of dislocations. Mechanical durability under high and inhomogeneous strain fields is an important aspect of exploiting Ag and Au NWs in applications like waveguiding or conductive networks in flexible polymer composite materials.

## Introduction

**Figure 1:**
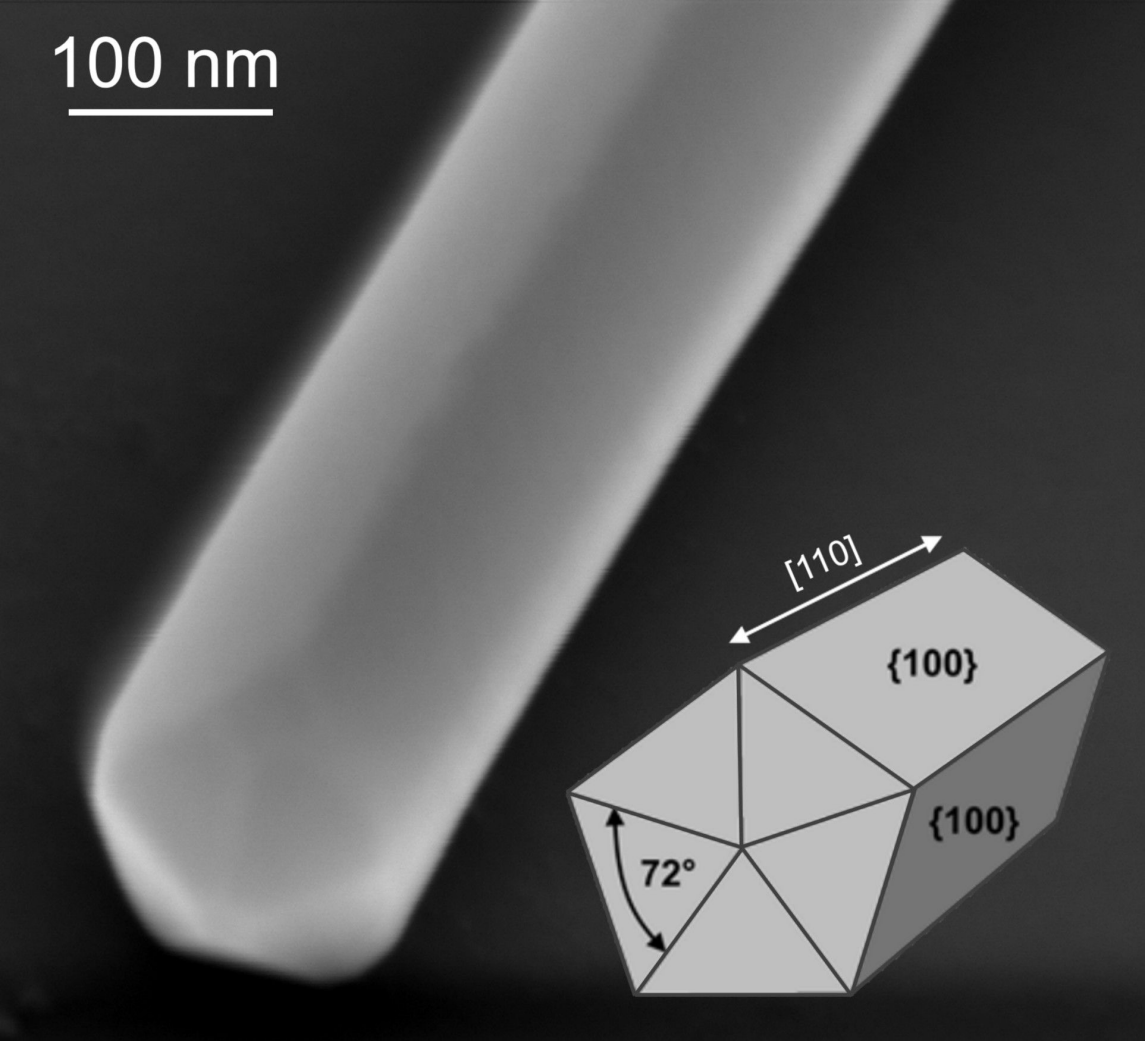
Schematic and SEM image of a pentagonal silver nanowire (Ag NW). SEM image adapted with permission from [28], copyright 2013 Elsevier.

Nanostructures comprised of noble metals with face centered cubic (FCC) crystal structure (Au, Ag and Cu according to the most common physical definition) prepared via soft chemical colloidal techniques often demonstrate a morphology with axes of five-fold (pentagonal) symmetry [1]. Depending on the synthesis conditions such structures can be synthesized in the form of 0D nanoparticles or high-aspect ratio 1D nanowires (NWs) with pentagonal cross-section [2–3]. The pentagonal NWs can be considered as 1D materials consisting of five prismatic monocrystalline domains with a triangular cross-section rotated relative to each other by approximately 72°, as shown schematically in Figure 1. The crystalline domains are divided by twin boundaries [4–5]. Due to the fact that the five fcc equilateral triangular segments connected by twin boundaries cannot make 360°, such an exotic structure cannot exist without internal strain with corresponding mechanical stress and stored elastic energy proportional to the volume [6].

Inner stress and peculiar structure of the pentagonal materials is expected to lead to mechanical properties different from those of regular monocrystals [7]. This fact must be taken into account when considering applications in which nanocrystals are subjected to mechanical deformation, for example, NW-based nanoswitches [8], nanoresonators [9] and flexible electronics [10–11]. In the last case NWs are used as a conductive network in composition with flexible polymer materials such as polydimethylsiloxane (PDMS) [12–13]. The reliability of flexible devices in high-strain conditions will be governed by the mechanical reliability of the individual NWs inside the conductive network [14–16]. Ag NWs are a promising material for flexible transparent electrodes [17]. Plasmon propagation and the optical properties of Ag and Au NWs make them attractive for nanophotonics as waveguides for visible light [18–23]. In all these applications, NWs may experience severe and sometimes repeated bending deformations. Therefore, the proper understanding of the mechanical behaviour of NWs under bending deformation and the use of appropriate theoretical models is essential for the design and function of NW-based devices.

The mechanical properties of pentagonal NWs were studied both theoretically and experimentally in different configurations, including uniaxial loading (tensile and compression tests) [24–25], three-point bending [26–27], cantilever beam bending [28–29] and nanoindentation [30]. Several interesting phenomena were reported that can be attributed to the peculiar inner structure of pentagonal NWs. Qin et al. reported on recoverable plasticity in penta-twinned Ag NWs [31] in tensile tests. The enhanced elasticity, exceptional strength and unexpected brittle failure of pentagonal Ag NWs were reported by Zhu et al. [25] in tensile tests and by Wu et al. [27] in three-point bending tests. According to the authors, the grain orientation and grain-boundary arrangement within the NWs are responsible for their exceptional strength and brittle-like fracture. The slip directions in the grains intersect with the twin boundaries, resulting in uniform structure hardening. Five-fold grain boundaries intersect with all possible slip systems restricting the motion of dislocations along any slip direction by the twin boundaries that extend to the center of the wire preventing the initiation of plastic deformation. This makes five-fold twinned NW grain-boundary-hardened material that sacrifices ductility for strength [27].

In both the tensile and three-point bending tests, the NW is rigidly fixed at both ends. Even though the NW is bent in the three-point bending test, the deflection of the NW before failure is relatively small in comparison to the so-called pure bending conditions when one of the NW ends is free. From the viewpoint of applications (e.g., waveguiding) the behavior of the NW under a pure bending condition, as opposed to tensile or three-point bending, is of great importance, as it is related to the ability of the NWs to form curved pathways for electromagnetic radiation. Any crack or other discontinuities that are introduced by bending can prevent plasmon propagation in the NW [19].

Pure bending conditions are realized in the cantilever beam bending configuration where the NW is fixed at one end and the free end is pushed by the probe. Such a configuration enables a high degree of bending. The behavior of the Ag NWs under pure bending conditions was studied experimentally by Vlassov et al. [28] in cantilever beam bending tests. Similar to Zhu and Wu [25,27], we noticed brittle-like fracture of the NWs in 1/3 of the cases. In 2/3 of the cases, plastic yield was reported. However, it should be noted that loading was applied in dynamic mode, i.e., the probe was oscillating at a frequency of around 32 kHz and the amplitude of the oscillations was comparable to the diameter of the NWs. Therefore, it is difficult to exclude the possible fatigue effect, indicating the need to perform cantilever beam bending tests in continuous loading mode.

In present work we performed bending tests on pentagonal Ag and Au NWs in a cantilever beam configuration inside a scanning electron microscope (SEM) for visual guidance. We demonstrate an unusual, abrupt elastic-to-plastic transition, observed as a sudden change of the NW profile from smooth arc-shaped to angled knee-like during the bending in the narrow range of bending angles (critical bending angle). Moreover, we show that if the NWs are coated with alumina, an abrupt plastic event is not observed and the NWs can withstand severe deformation in the elastic regime without fracture.

## Materials and Methods

**Nanowires:** Ag NWs were purchased from Blue Nano (USA). Au NWs were purchased from Smart Materials (Latvia) [32].

**SEM and TEM characterization:** The micrographs of the NWs were obtained with a high-resolution scanning electron microscope (HR-SEM, Helios Nanolab 600, FEI) and transmission electron microscope (TEM, Tecnai GF20, FEI). The NWs were drop-cast on either TEM grids with lacey carbon (Agar, UK) for TEM characterization, or on glassy carbon and silicon wafers (100, n/phosphorus doped, 3–6 Ω·cm, Mat-Technology) for SEM imaging.

**Experimental set-up for bending tests:** The NWs were drop-cast on TEM grids (Agar) so that some of the NWs were partially suspended over a hole. The bending tests were performed inside a HR-SEM (Helios Nanolab 600, FEI) with a cantilever beam-bending configuration in a similar manner as described in [33–34]. The NWs were bent in-plane with a substrate by an atomic force microscope (AFM) probe (ATEC−CONT cantilevers, Nanosensor, Neuchatel, Switzerland, *C* = 0.2 N·m^−1^) attached to a micromanipulator (MM3AEM, Kleindiek, Germany).

**FEM simulations:** The cantilevered beam bending experiments were simulated using the finite element method (FEM) with COMSOL Multiphysics 5.2 solid mechanics module. For this the linear elastic material model from COMSOL was chosen. The simulations were based on a recently developed segmented pentagonal NW model [29]. In this model, the pentagonal NW was composed of five triangular prism-shaped domains with vertex angle of 72°. These domains represent the FCC single crystals. Each domain was assigned an elasticity matrix of Ag or Au corresponding to their crystal structure to account for structural anisotropy. The elasticity matrix independent parameters in Voigt notation for Ag were *C*_11_ = 124 GPa, *C*_12_ = 93.4 GPa, *C*_44_ = 46.1 GPa and for Au *C*_11_ = 190 GPa, *C*_12_ = 161 GPa, *C*_44_ = 42.3 GPa. The NW was fixed by a portion of the bottom facet at one end and pushed at the other end by applying gradually increasing force. The mesh used in these simulations is described in previous work [29].

The yield strength values were obtained from the FEM NW model by fitting its profile to the experimentally bent profiles of Ag or Au NWs at the critical bending angle, before the abrupt transition to plastic deformation.

**Figure 2:**
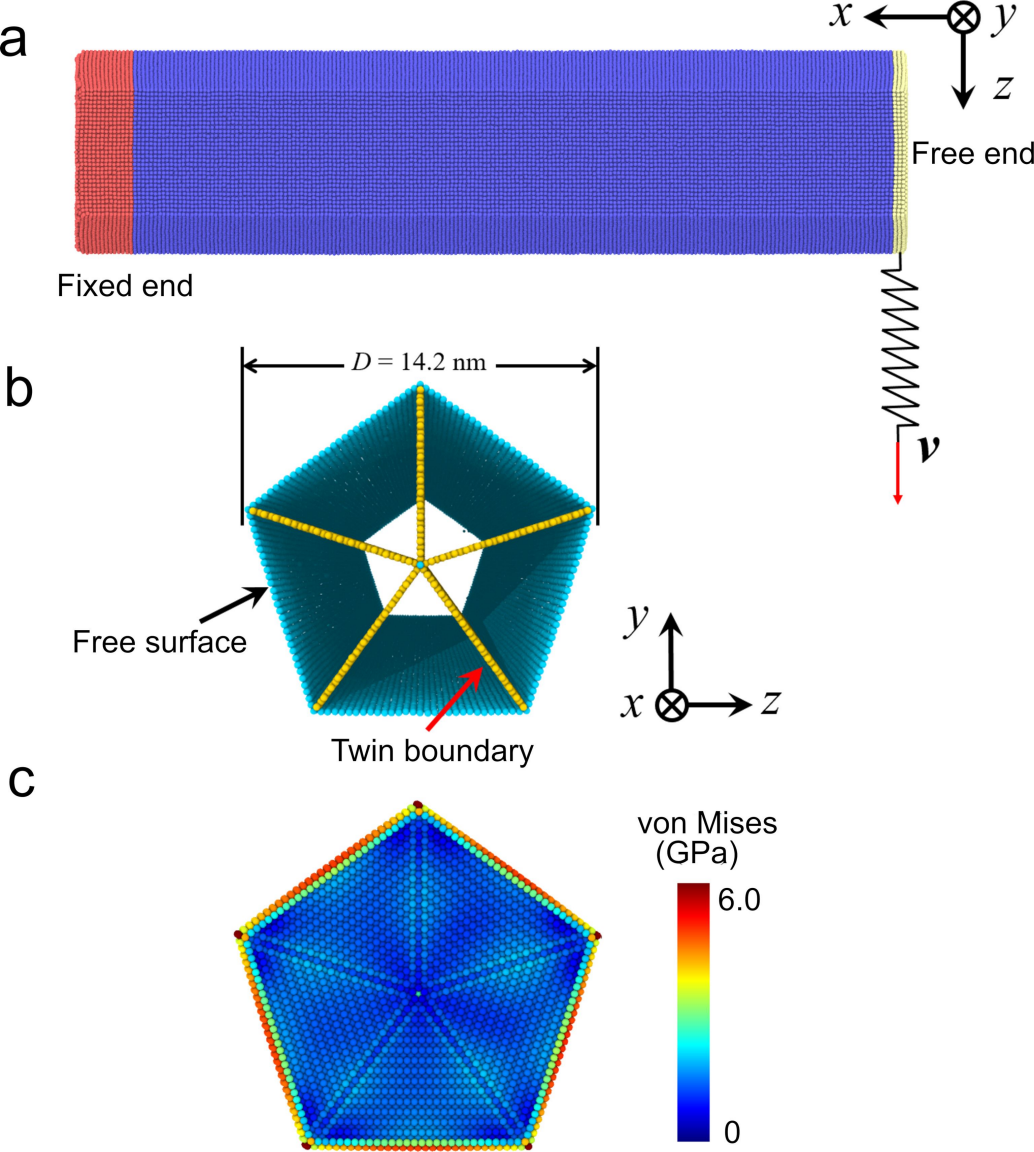
(a) Atomic simulation model of the Ag NW, (b) the internal twin structures (atoms in perfect lattice and those at the two ends are not shown for clarity), and (c) the distribution of von Mises stress on the cross section.

**MD simulations:** Classical molecular dynamics (MD) simulations were conducted to investigate the atomic deformation behavior of the penta-twinned Ag NW under bending. The large-scale open-source molecular dynamics simulator, LAMMPS, developed by Sandia National Laboratories, was adopted [35]. The interatomic interactions are described by the widely used embedded atom method (EAM), and a potential for Ag is utilized here [36]. Due to the limitation of computational resources, the size of the nanowire in simulation is much smaller than that in the experiments. Figure 2a shows the atomic configuration of the penta-twinned NW in the present simulation. The simulated model is ≈60.0 nm in length and ≈14.2 nm in diameter. The total number of Ag atoms is ≈0.474 million. Figure 1b shows the initial five-fold internal twin structures. In the simulation, free boundary conditions are imposed in all directions. The time evolution of atoms are within the framework of canonical (NVT) ensembles, and the time step is chosen as 2.0 fs. The Nosé–Hoover thermostat is used to adjust the temperature of the atomic system to around 300 K [37–38].

After construction of the NW, structural relaxation is firstly performed by using a conjugated gradient method in order to make the configuration reach a local stable state. The NW is further equilibrated at 300 K for about 40 ps to relax the internal stress. Figure 1c shows the distribution of von Mises stress on the cross-section after this equilibration. To apply a bending load to the NW at the fixed end, atoms with a layer thickness of 4 nm (the part colored in red in Figure 2a) are kept and fixed in a perfect lattice. At the free end, atoms with a layer thickness of 1.0 nm (the part colored in yellow in Figure 2) are kept rigid and will move as a whole. The rigid layer is then coupled to a spring with stiffness of 30.0 N/m. In the loading procedure, the free end of the spring moves at a constant speed of 0.05 Å/ps, and the NW will be bent by the spring. We monitor the bending force exerted by the spring to the NW and the displacement of the rigid free end to characterize the loading response of the NW.

## Results and Discussion

**Figure 3:**
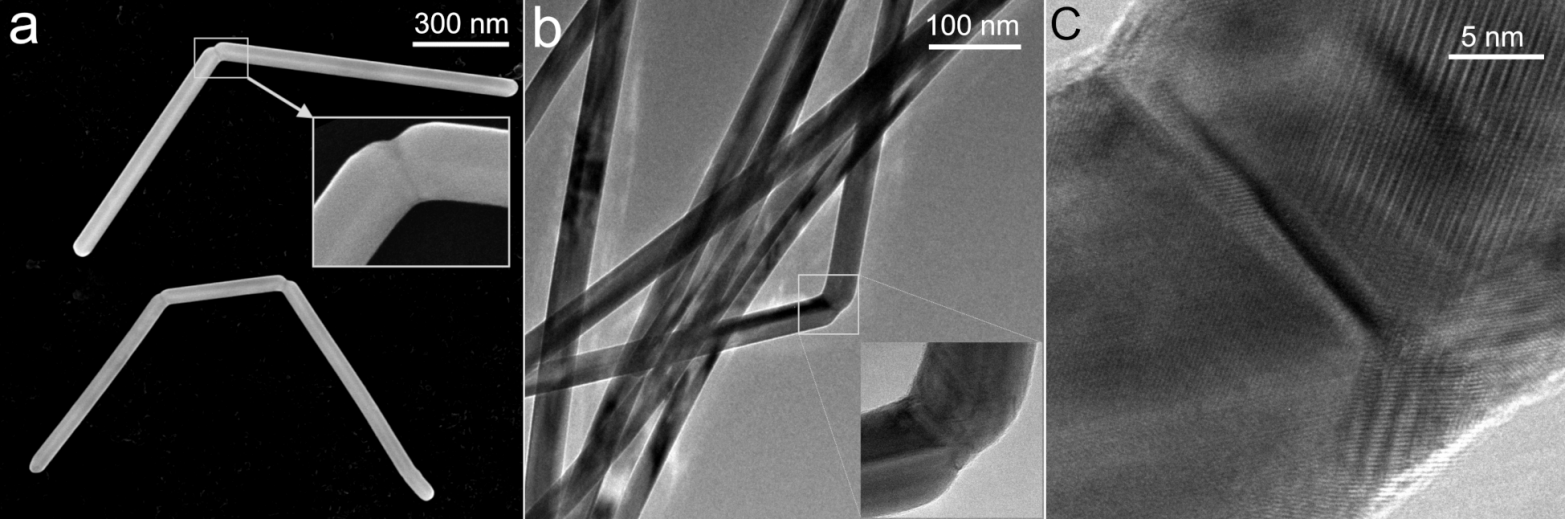
SEM (a) and TEM (b,c) images of deformed Ag NWs drop-cast from a solution.

According to the SEM and TEM images, both the Ag and Au NWs had a uniform diameter with a pentagonal cross-section and well-pronounced facets. The diameters ranged from several tens of nanometers to over 100 nm with lengths ranging from several hundreds of nanometers to tens of micrometers. Some of the NWs had a clearly pronounced angled profile that resembled some sort of a so-called “greenstick fracture” (Figure 3). Visually very a similar phenomenon was reported for Ag NWs by Peng et al. [39] and explained as the joining of two adjacent NWs. In the present work, we challenged the explanation given by Peng et al. and performed in-situ SEM bending and breaking of Ag in order to determine if the visible profile of the NWs is related to the brittle-like mechanical deformation rather that the joining of two NWs together. Experiments were also performed on Au NWs to determine if the observed phenomena is material-dependent or rather related to the inner five-fold twinned structure.

**Figure 4:**
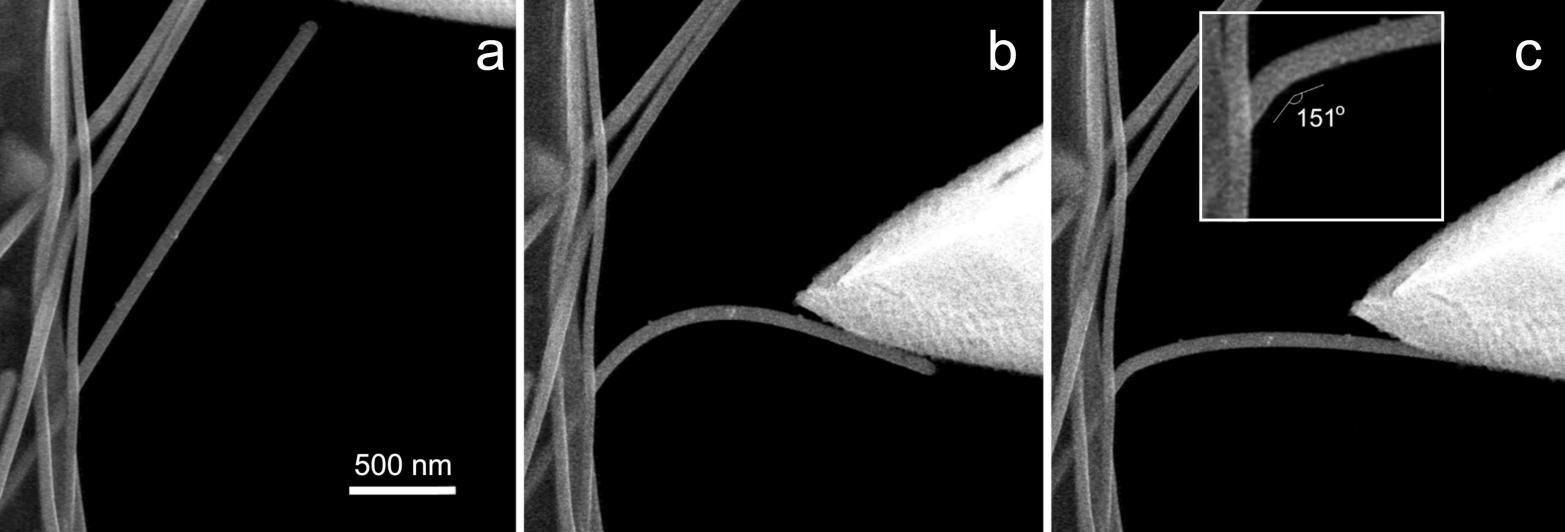
Ag NW bending experiment observed inside a SEM. (a) Straight NW before bending, (b) NW bent close to the critical bending angle, (c) abrupt elastic-to-plastic transition occurred.

When either Ag or Au NWs were gradually bent by a sharp Si tip in a cantilever beam configuration inside the SEM, an abrupt elastic-to-plastic transition event was revealed at a critical bending angle. Namely, at the beginning of the bending tests, the NWs exhibited extensive elastic behavior, which is common for one-dimensional nanostructures [40]. However, at a certain curvature, the bending profile of the NW suddenly changed from smooth arc-shaped to an angled knee-like bend (Figure 4). This behavior was general for both Ag and Au NWs. This finding was not trivial, since macroscopic Ag and Au undergoes gradual plastic deformation under loading. In our case the NWs underwent an abrupt event of plastic deformation to a new crystallographic configuration. This new configuration was elastically stable, that is, if the “angled” NW was moderately bent by the AFM tip either further or straightened and then released, it went back to its initial angled profile. Moreover, a complete fracture was difficult to achieve in such configuration, even under severe deformation.

**Figure 5:**
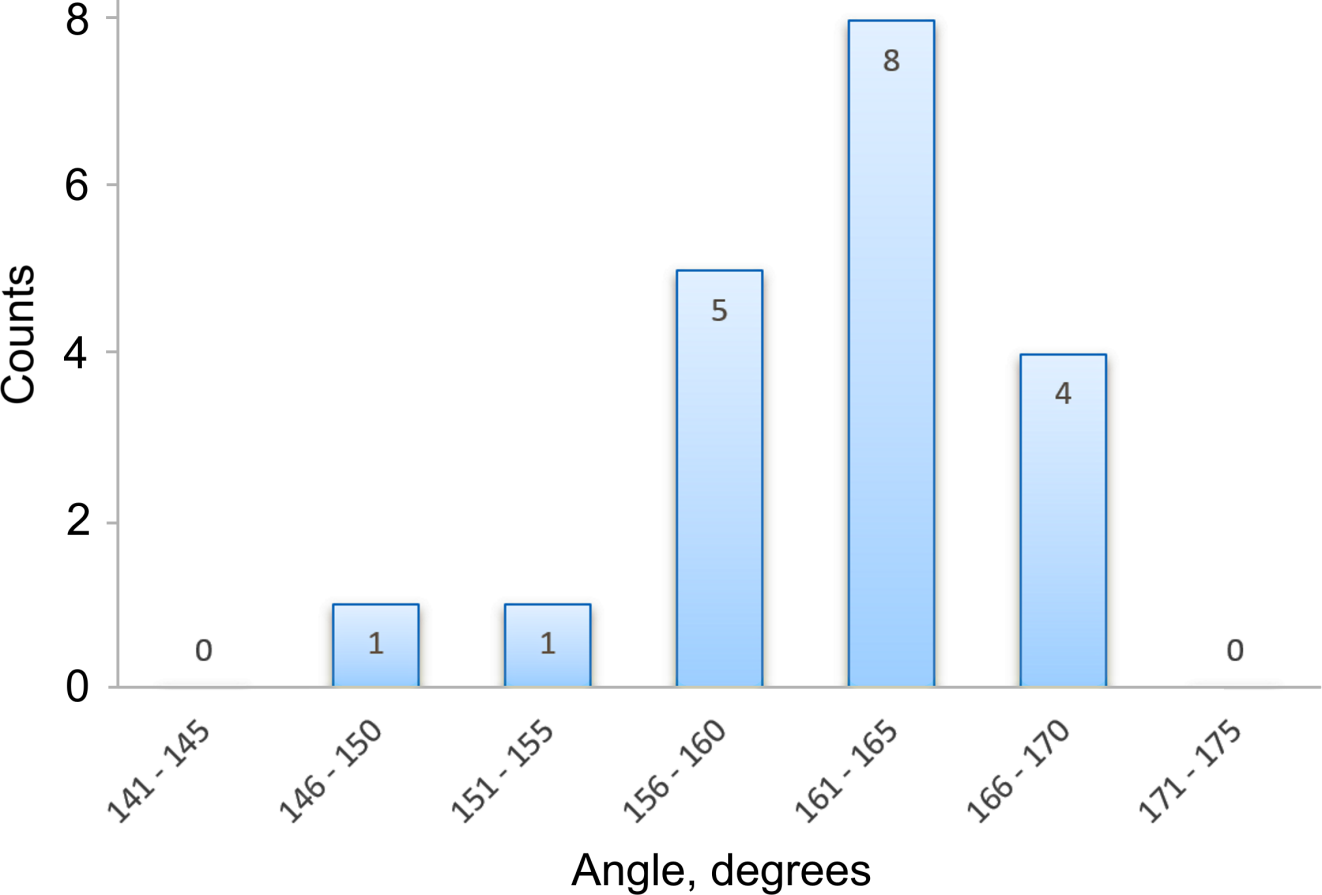
Distribution of angles of the deformed NWs after an abrupt plastic event in the bending tests.

In total 19 (6 Ag and 13 Au) NWs were tested and it was found that the angle of the deformed NWs (after the initial plastic event) lies in the narrow range of 151–169° for both Ag and Au with a median value of 163 ± 5.6° (Figure 5). This fact indicates that the observed abrupt elastic-to-plastic transition is not a random process but is closely related to the inner structure of the five-fold twinned NWs.

In order to obtain yield strength values and to have a clearer understanding regarding stresses present in the NW during yielding, an FEM model was developed that included the inner anisotropy of five-fold twinned NWs. The geometrical parameters and maximal deformation of the NWs at the critical bending angle (prior transition) were taken from the real experiments.

**Figure 6:**
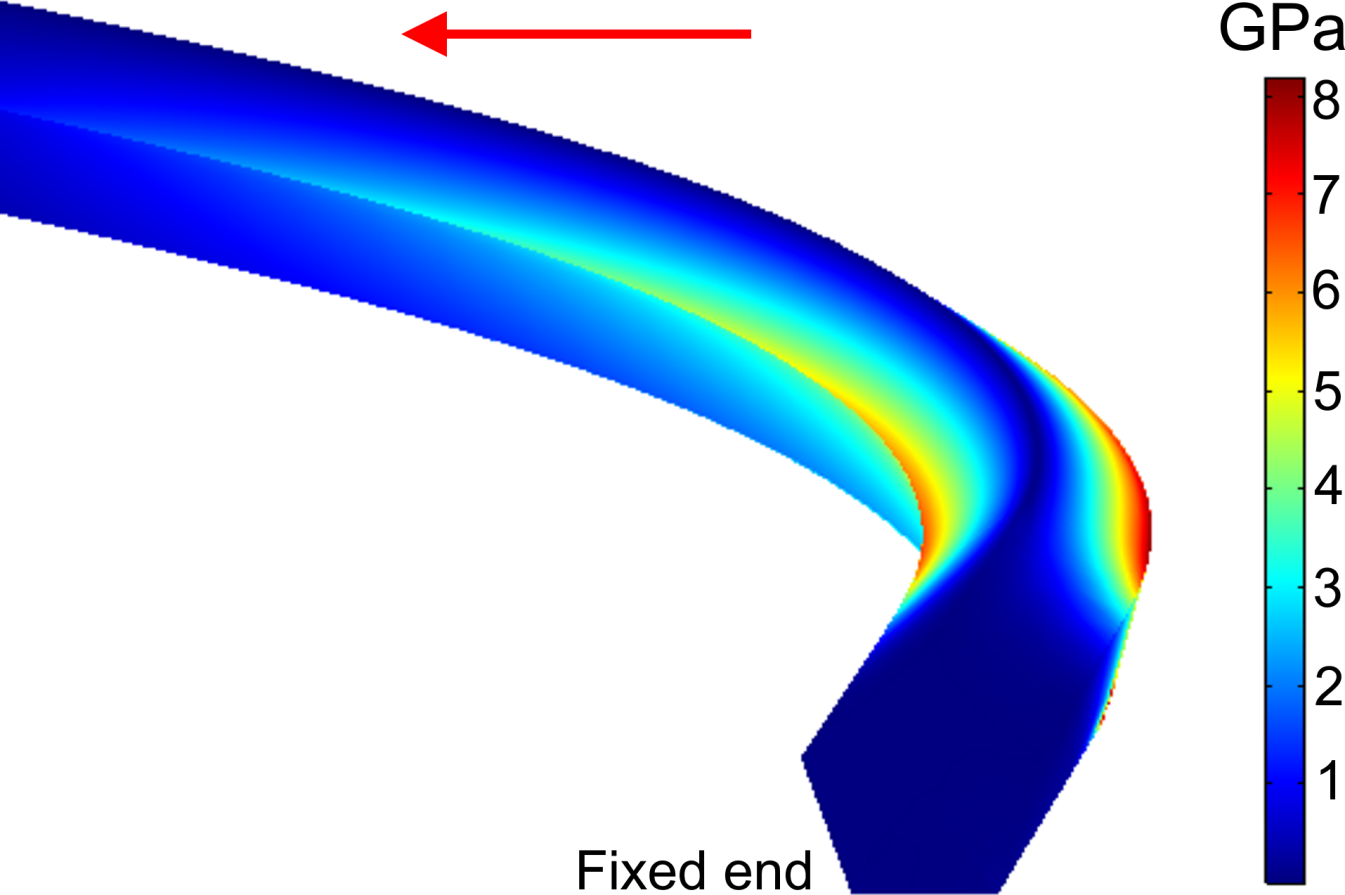
Von Mises stress distribution in a bent Au NW at the critical bending angle, corresponding to the maximal experimental curvature prior to fracture (GPa).

**Figure 7:**
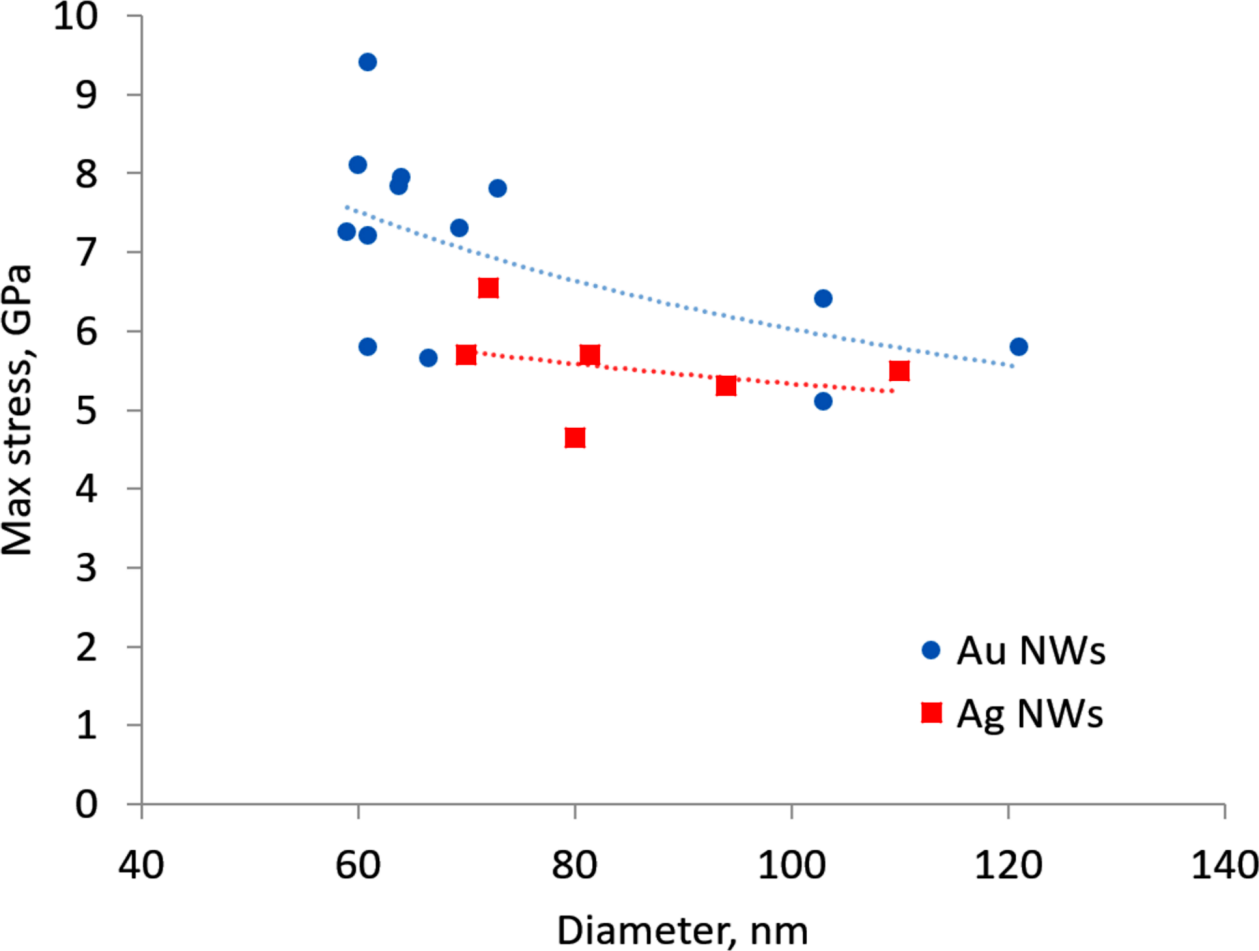
Au and Ag NW yield strengths as revealed from the bending tests.

The von Mises stress distribution for the FEM-simulated bent NW is shown in Figure 6. The stresses are concentrated mostly on two edges of the bent NW near the fixed part. The highest stress values are on the outer edge in the range of 4.65 to 6.54 GPa for Ag NWs and from 5.1 to 9.4 GPa for Au NWs (Figure 7). The median yield strength values for Ag and Au NWs were 5.6 GPa and 6.8 GPa, respectively. The tendency of increasing strength for smaller diameters (size effect) can be noticed.

For Ag NWs, the results are in good accordance with experimental yield strength values obtained for Ag NWs using the similar cantilever beam configuration [28]. It should be noted that measurements in [28] were performed in dynamic mode, that is, the probe and hence the NW were oscillating during the measurements with amplitude on the order of 100 nm. Moreover, the experiments were performed in a lower resolution SEM. Therefore, if an abrupt transition took place, it was left unnoticed. However, the sudden slope change on the force–displacement curve in Figure 5 in [28] can be explained by the sudden transition from elastic-to-plastic deformation.

To our knowledge, no experimental works determining the strength of pentagonal Au NWs have been published yet; therefore a direct comparison cannot be made. Nonetheless, the values obtained in the present work are comparable, but do not exceed the theoretical yield strength value of bulk Au, which is about 8 GPa in accordance with the empirical relation E/10 based on atomic bonding considerations [41].

### Molecular dynamics results

**Figure 8:**
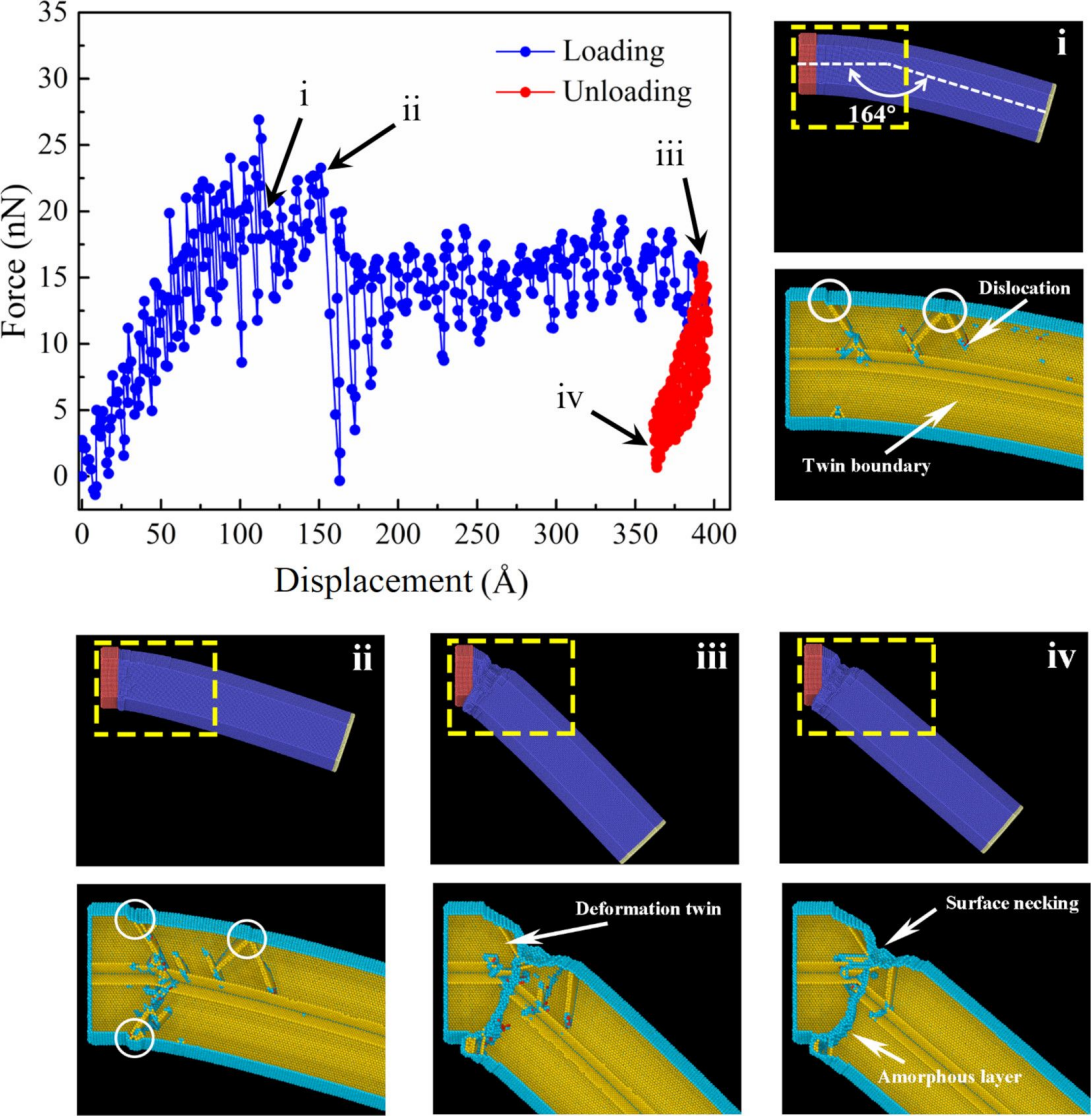
Bending force versus displacement curve (top) and the evolution of the atomic structure and the internal defects during bending of the NW (bottom). In the local cross-section of the NW, atoms in a perfect lattice are not shown for clarity.

Figure 8 depicts the loading curve of the penta-twinned NW (link to video in Supporting Information File 1). Owing to the influence of temperature and the natural frequencies of the atomic system, the NW vibrates during the loading procedure, leading to the oscillation of the bending load. When referring to the loading curve, we only consider the whole trend. In the elastic regime, the loading force linearly increases with the displacement. When yielding occurs, the load drops abruptly. We examined the atomistic structure of the NW immediately after the load peak. The bending angle of the NW at the abrupt elastic-to-plastic transition is ≈164°, which agrees very well with the transition angles 163 ± 5.6° observed in the experiment. Dislocations nucleate from the free surface as marked by the white circles (Figure 8i). After nucleation, partial dislocations glide toward the core region of the NW and experience the impediment from the existing twin boundaries [42]. With the progression of bending deformation, another dislocation source is also activated, and a new dislocation nucleation and propagation lead to the second distinct load drop of the loading curve (Figure 8ii). In the following stage, the bending force gradually becomes stable at approximately 15 nN, and the dislocations continue to nucleate in the region close to the fixed end. Besides the existing twin boundaries, a deformation twin could also be observed (Figure 8iii). The interaction of continuously nucleated dislocations will form an amorphous layer, similar to a grain boundary. With the increased concentration of the deformations surface necking occurs, which will lead to the ductile fracture of the NW. Upon unloading, some dislocations may retract and annihilate at the free surface, which is promoted by the existing twin boundaries [31] and the amorphous atomic layer and surface necking remains (Figure 8iv).

In order to confirm if the deformation is rate dependent, we also performed a quasi-static simulation. The results are shown in Figure S2 in Supporting Information File 1. The deformation of the NW was similar to that in the main result and is rate-independent. The stress distributions in the NW cross-section near the fixed end at loading rate of 0.05A/ps is shown in Figure S3 in Supporting Information File 1.

In rare cases the slip of dislocations across the twin boundary was observed (Figure S4). This is due to the fact that the corners of the NW are preferential dislocation nucleation sites because of the stress concentration, and therefore deformation is easier to sustain by new dislocation nucleation rather than cross-slipping of existing dislocation at twin boundaries.

On the basis of the conducted experiments, electron microscopy observations and simulations we can speculate about possible reasons behind the observed phenomena. The abrupt elastic-to-plastic deformation transition of the FCC pentagonal NWs under cantilevered beam bending can be attributed to their inner structure. The plastic deformation in metals is the result of the formation and propagation of dislocation defects. For the pentagonal NWs, the propagation of these defects is hindered by the five twin boundaries inside these structures, leading to the accumulation of dislocations at the twin boundaries [43]. In the cantilevered beam bending configuration, this accumulation is more pronounced due to asymmetric deformation. This dislocation pile up can result in increased strength and may lead to sudden stress release, which is observed as an abrupt plastic event.

**Figure 9:**
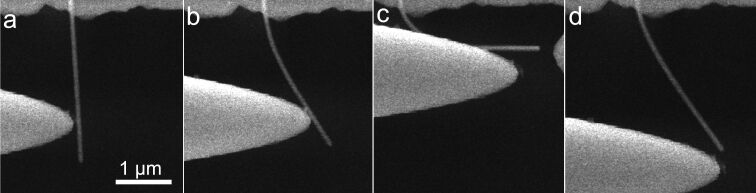
Bending of an alumina-coated silver NW. No breaking or abrupt deformation were observed even for relatively high bending curvatures.

The surface nucleation of dislocation in nanoscale objects such as NWs might play a major role in plastic yield due to their extreme surface-to-volume ratio. If surface nucleation is mitigated by, for example, external materials (coating), the onset of plastic yield can be significantly postponed. Hence, we performed a few preliminary tests on Ag NWs coated by atomic layer deposition (ALD) with a few-nm thick layer of alumina (Figure 9). An abrupt elastic-to-plastic transition was not observed even for bending curvatures up to 90°. A hard coating may prevent stress release mechanisms related to dislocation pile up and dislocation nucleation at the surface [33–34]. However, more systematic studies on the role of coating need to be conducted and this will be addressed in our future research.

## Conclusion

We demonstrated an abrupt elastic-to-plastic transition event for Ag and Au NWs in cantilever beam bending tests inside an SEM. The event consisted in an abrupt transition from an arc-shaped profile to an angled profile in the narrow range of angles 163 ± 5.6° measured on 19 NWs. The maximal stress values in the most bent state prior to the transition were 5.6 GPa and 6.8 GPa for Ag and Au NWs, respectively, which is close to the theoretical strength of this materials. The abrupt transition can be attributed to two factors: the inner structure of the pentagonal NWs and its free surface. The five twin boundaries dividing the structure can hinder the propagation of dislocations and prevent a smooth onset of plastic deformation under a cantilevered beam bending configuration. Therefore, the accumulation of dislocations at the twin boundaries leads to the brittle fracture-like onset of plastic deformation. The free surface of the NW facilitates the nucleation of defects, such as dislocations in an otherwise practically defect-free pentagonal NW structure. Additional preliminary experiments with alumina-coated Ag NWs showed that coating can increase the bending strength of the NWs.

## Supporting Information

File 1 Additional figures and a video link.
