# Supplementary material for: Abrupt elastic-to-plastic transition in pentagonal nanowires under bending
Source: Beilstein J Nanotechnol. 2019 Dec 12;10:2468–76. doi: 10.3762/bjnano.10.237 (PMC6941403; doi:10.3762/bjnano.10.237)
Supplement: File 1 — Additional figures and a video link. [file Beilstein_J_Nanotechnol-10-2468-s001.pdf]

BEILSTEIN JOURNAL OF NANOTECHNOLOGY

Supporting Information

for

Abrupt elastic-to-plastic transition in pentagonal nanowires under bending

Sergei Vlassov, Magnus Mets, Boris Polyakov, Jianjun Bian, Leonid Dorogin and Vahur Zadin

Beilstein J. Nanotechnol. **2019,** *10,* 2468–2476. doi:10.3762/bjnano.10.237

Additional figures and a video link

License and Terms: This is a supporting information file under the terms of the Creative Commons Attribution License (https://creativecommons.org/licenses/by/4.0). Please note that the reuse, redistribution and reproduction in particular requires that the authors and source are credited. The license is subject to the *Beilstein Journal of Nanotechnology* terms and conditions: (https://www.beilstein-journals.org/bjnano)

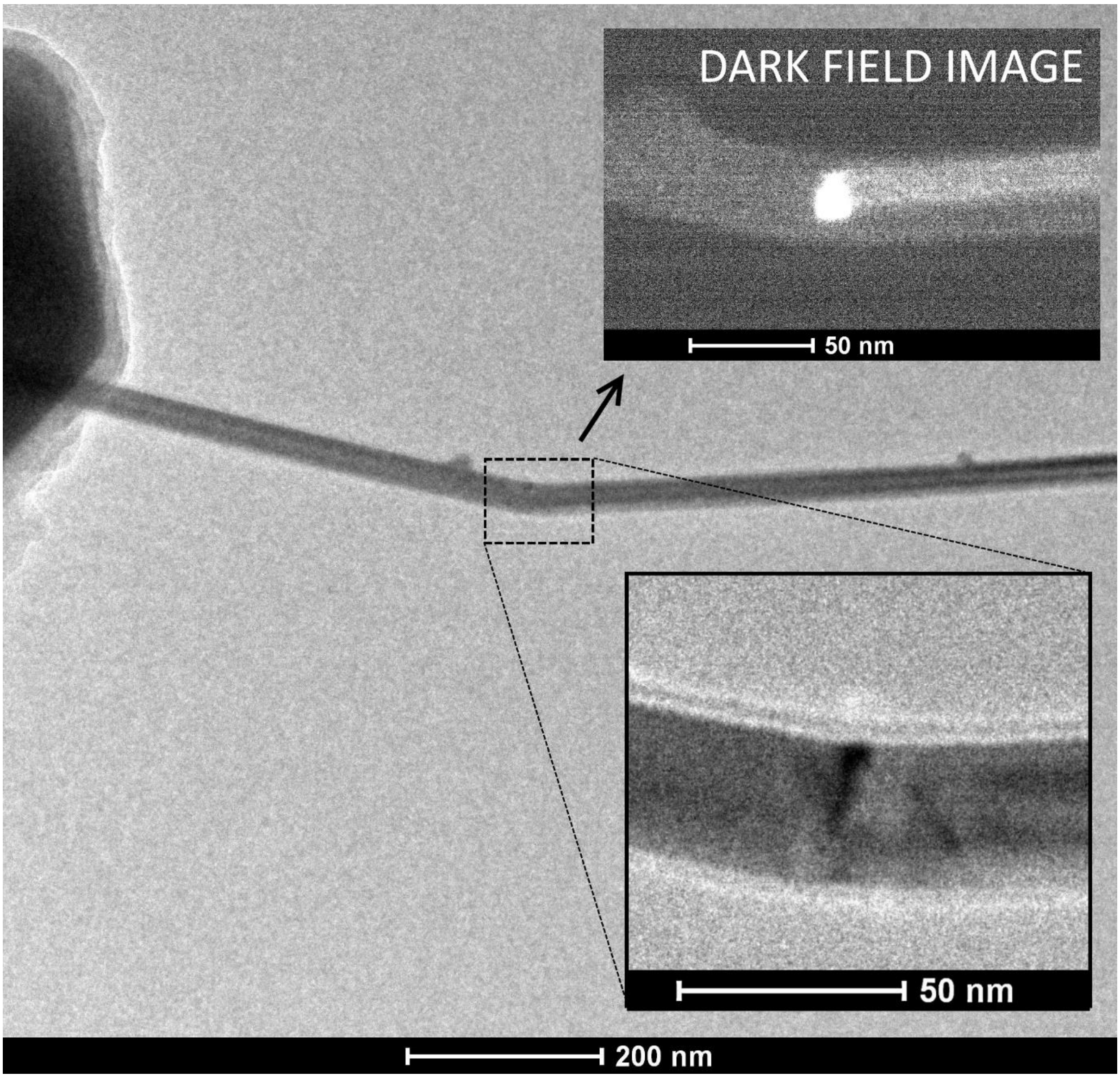

Figure S1: Bright and dark field TEM images of Ag nanowire previously bent inside SEM by nanomanipulator.

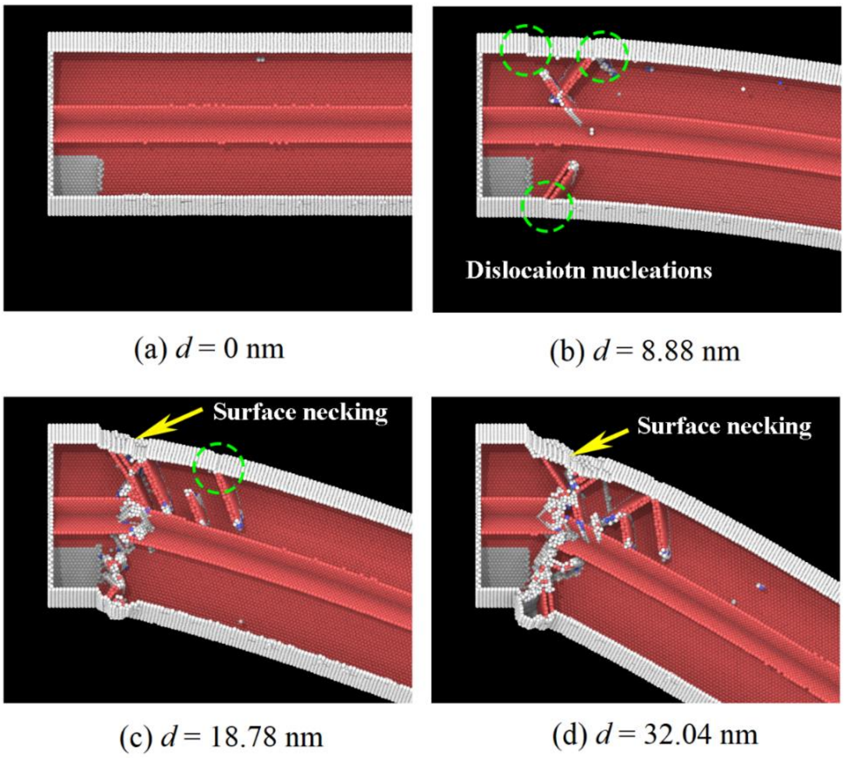

Figure S2: Dislocation structures of the Ag fivefold nanowire in a quasi-static simulation. (Red atoms are twin boundaries and stacking faults, while atoms are dislocation cores and free surfaces. Atoms in perfect lattice are not shown for clearness).

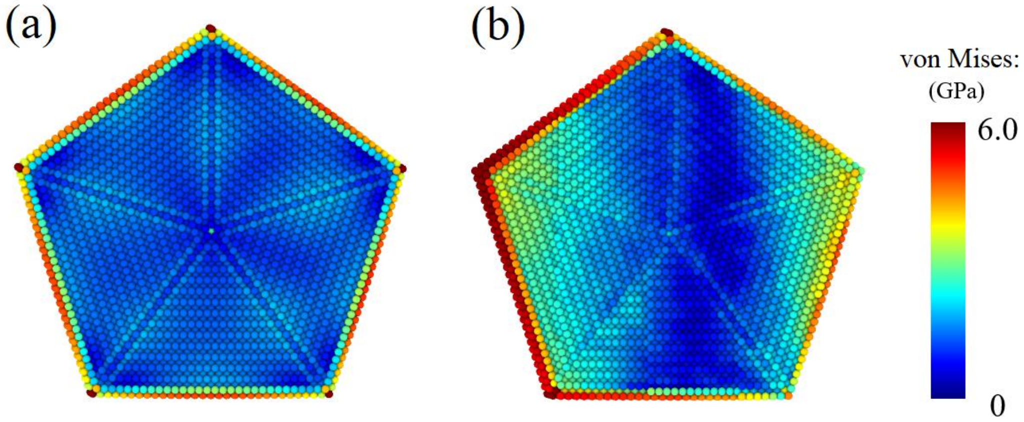

Figure S3: Stress distribution of the Ag nanowire at the initial stage (a) and the stage before the first dislocation nucleation (b).

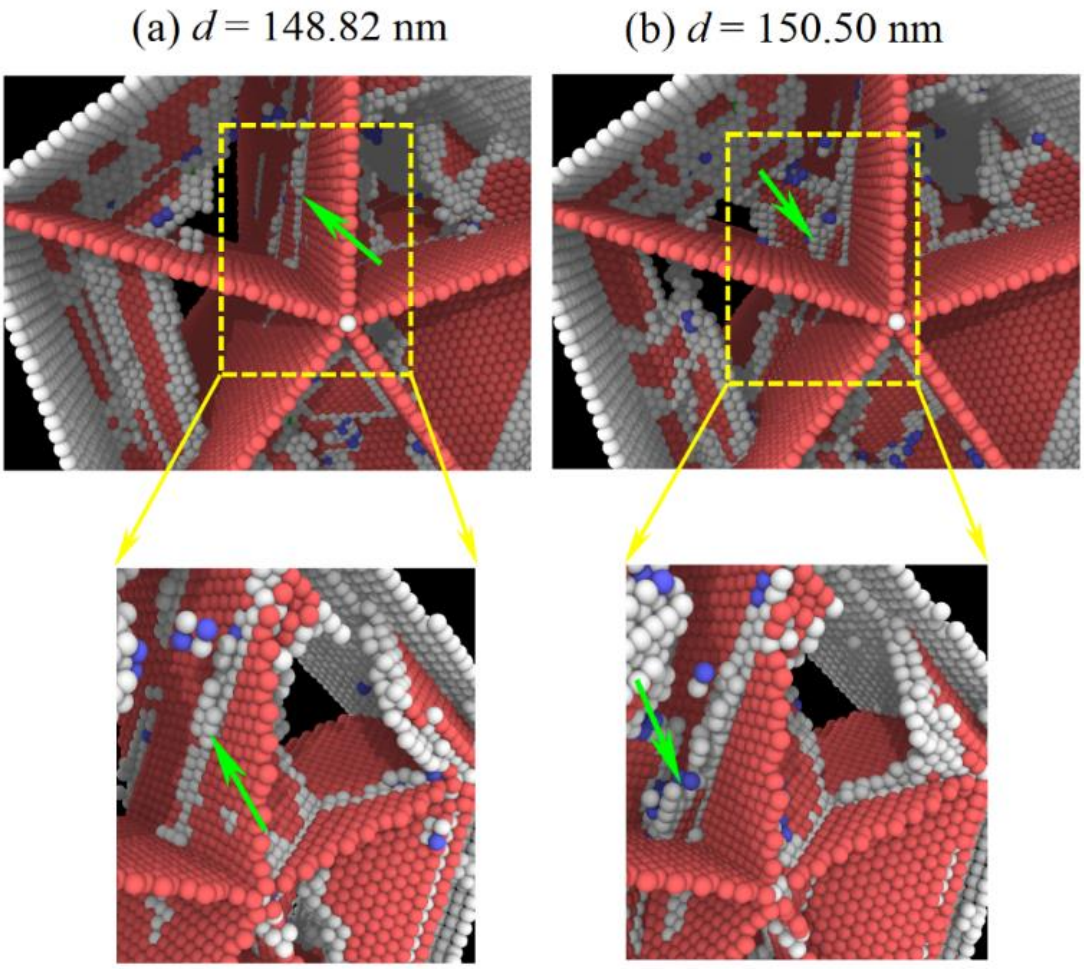

Figure S4: Dislocation cross-slip a twin boundary. (Atom color scheme is the same to that in Figure S2)

Video: Bending of pentagonal Ag nanowire, MD simulations.

https://1drv.ms/u/s!AuJGTOfjxvz2hAjtbDQKcvr-yY4f?e=kyPIcF
